# Circulating miR-29b decrease in response to sarcopenia in patients with cardiovascular risk factors in older Chinese

**DOI:** 10.3389/fcvm.2022.1094388

**Published:** 2022-12-20

**Authors:** Nana He, Yuelin Zhang, Yue Zhang, Beili Feng, Zaixing Zheng, Honghua Ye

**Affiliations:** ^1^Medical Data Center, Ningbo City First Hospital, Ningbo, Zhejiang, China; ^2^Key Laboratory of Precision Medicine for Atherosclerotic Diseases of Zhejiang Province, Ningbo, Zhejiang, China; ^3^Department of Cardiology, HwaMei Hospital (Previously Named Ningbo No. 2 Hospital), University of Chinese Academy of Sciences, Ningbo, Zhejiang, China; ^4^Department of Cardiovascular, Lihuili Hospital Facilitated to Ningbo University, Ningbo, Zhejiang, China

**Keywords:** circulating microRNAs, sarcopenia, biomarker, CVRF, elderly

## Abstract

**Introduction:**

Sarcopenia is a clinical syndrome characterized by a progressive and extensive decline in skeletal muscle mass, muscle strength, and function. Sarcopenia and cardiovascular diseases (CVDs) can coexist, which further decreases the quality of life of patients, and increases the mortality rate. MicroRNAs (miRNAs) are unique posttranscriptional regulators of gene expression whose function in aging-related sarcopenia and CVDs has recently begun to unravel. The aim of the present study is to investigate the relationship between sarcopenia and cardiovascular risk factors (CVRF) in the Chinese elderly and describe the circulating miRNAs in sarcopenia patients with the intention of identifying novel diagnostic and therapeutic tools.

**Methods:**

The well-established CVRF of diabetes, hypertension, and dyslipidemia were assessed. Multiple logistic regression analyses and linear regressions were used to evaluate the components of CVRF and the number of CVRF in elderly patients with sarcopenia. Moreover, we used real-time RT-PCR to measure the abundance of the CVRF-related miRNAs in the plasma of a cohort of 93 control and sarcopenia individuals, including miR-29b, miR-181a, and miR-494.

**Results:**

We found that CVRF was associated with a high prevalence of sarcopenia in elderly Chinese populations After adjusting for potential confounders. Furthermore, hypertension and dyslipidemia, but not diabetes, were found to be significantly associated with sarcopenia. A linear increase in the prevalence of sarcopenia was found to be associated with the number of CVRF components in the elderly population. We found that plasma miR-29b levels were significantly down-regulated in response to sarcopenia in the elderly with CVRF. In particular, there was a remarkable correlation between miR-29b and appendicular skeletal muscle mass (ASM)/height2. Collectively, knowledge of CVRF, particularly hypertension and dyslipidemia, may help predict the risk of sarcopenia in the elderly. Our data also show that circulating miR-29b can be considered as possible biomarkers for sarcopenia, which may also be used in the CVD assessment of these patients.

**Discussion:**

We found that the prevalence of sarcopenia was significantly proportional to the number of CVRF components. In particular, hypertension and dyslipidemia were significantly associated with a higher risk of sarcopenia in the adjusted models. Moreover, our study has been proven that c-miRNAs may be considered as possible biomarkers for sarcopenia as a new diagnostic tool to monitor response to treatment. There is also a pressing need for further research on sarcopenia and CVRF to understand their relationship and mechanism. These can provide more evidence to develop potential interventions to improve clinical outcomes.

## Introduction

Sarcopenia is a disease characterized by decreased skeletal muscle mass, muscle strength, and physical performance. It is related to the aging process, which can lead to significant morbidity and disability in the elderly population, including reduced poor physical function, loss of independence, poor quality of life, falls, and mortality (1). A number of consensus groups have developed algorithms to identify sarcopenia based on the combination of muscle function loss and mass loss over the past decade (2–5). Subsequently, there has been a rapid increase in research interest in sarcopenia around the world, including Asia (5). It was indicated that the prevalence of sarcopenia among the elderly is expected to increase significantly in the future. Globally, it is estimated that approximately 50 million people are suffering from sarcopenia, and the number is expected to reach 500 million by 2050 (6). In Asia, sarcopenia is prevalent among older adults at a rate ranging from 6.8 to 25.7% (7). Consequently, there is a significant increase in the current cost of healthcare for sarcopenia, which has led to a major public health problem. As a result of the debilitating symptoms of sarcopenia and the increased cost of medical treatment, it is important to prevent and diagnose it at an early stage. Early detection and intervention of risk factors for sarcopenia is of great significance to improving the quality of life of the elderly, reducing complications and avoiding serious consequences. Nevertheless, the current clinical diagnostic criteria for sarcopenia are different greatly, and there are few biological markers of sarcopenia, which has led to significant challenges and difficulties in the prevention and treatment of sarcopenia.

The pathogenesis of sarcopenia is very complex and there are multiple interactions. The mechanisms responsible for sarcopenia have been extensively studied, including decreased physical activity levels, hormone deficiencies, decreased protein intake, dyslipidemia, insulin resistance, and chronic inflammation that results in the release of catabolic cytokines. Reasonable physical exercise and a balanced diet are the most effective ways for older adults to prevent muscle function decline (8–11). At present, the most widely reported mechanism of sarcopenia is chronic inflammation and the production of catabolic cytokines (12, 13). It is thought that the development of sarcopenia is due to normal aging, but it has multiple causes. These causes include diabetes, obesity, inactivity, hypertension, and dyslipidemias (14). Among these factors, diabetes, hypertension, and dyslipidemia are well known to be risk factors for cardiovascular disease (CVD) (15, 16).

Cardiovascular disease is one of the leading causes of death throughout the world, and its prevalence is on the rise in China (17). Recent decades have seen an exponential increase in the prevalence of its associated cardiovascular risk factors (CVRF). Sarcopenia is a risk factor for hyperlipidemia, dyslipidemia and insulin resistance, which increases the risk of CVD. According to a cross-sectional study, sarcopenia is associated with an increased risk of CVD, which may lead to an increased number of incident cardiovascular events (18). To the best of our knowledge, no studies have examined the relationship between possible sarcopenia and incident CVD. Additionally, few studies have been conducted to determine the relationship between sarcopenia and CVRF (19).

MicroRNAs (miRNAs) have increasingly become potential markers of disease with the advancement of precision medicine and next-generation sequencing. It is an endogenous, conserved, single-stranded molecule composed of about 22 small non-coding RNAs, which can regulate the expression of target genes after transcription and has been shown to be involved in various biological processes (20, 21). Changes in miRNA expression levels can be a response to assessing human health status since miRNAs are stable and abundant, and can be easily detected in blood circulation (e.g., plasma). As a new, more sensitive, and potentially new biomarker for disease diagnosis and treatment, circulating miRNAs (c-miRNAs) have been reported to have a signature for patients with sarcopenia (22, 23). The level change of one of these CVRF-associated miR-29b (24, 25), miR-181-5p (26–28), and miR-494 (29–31) has been predicted to be related to patients with sarcopenia more recently. Considering that sarcopenia and CVD are closely related to CVRF, we can reasonably assume that sarcopenia can be assessed by measuring CVRF-specific c-miRNAs which can reflect health status. However, it is still unclear how circulating miRNA levels change in patients with sarcopenia.

Finally, considering the association between sarcopenia and CVD in older people and the absence of appropriate and consistent evidence regarding the association between sarcopenia and CVRF, we conclude that further research is needed to investigate the relationship between sarcopenia and CVRF to improve public health in China. In this study, we examined the effects of the prevalence of different numbers of CVRF components on sarcopenia using the AWGS definition in suburb-dwelling populations of elderly persons aged 65 and older in China. We found that the prevalence of sarcopenia was significantly proportional to the number of CVRF components. In particular, hypertension and dyslipidemia were significantly associated with a higher risk of sarcopenia in the adjusted models. Moreover, we determined the change of the well-functioning specific circulating miRNAs respond to sarcopenia in the elderly. Our data showed that plasma miR-29b levels were significantly downregulated in response to sarcopenia in elderly, while miR-181a and miR-494 were not changed significantly. We also correlated the circulating miRNA changes with diagnostic indicators of sarcopenia [appendicular skeletal muscle mass (ASM)/Height2, Handgrip strength, and 4-m velocity], showing the potential of circulating miRNA as biomarkers of sarcopenia in elderly.

## Materials and methods

### Ethics statement

This research was approved by the Ethics Committee at Ningbo No. 2 Hospital (PJ-NBEY-KY-2016-020-01), and it was conducted according to the principles of the Declaration of Helsinki. All records have been anonymized, and no individual information can be identified. All participants were informed in advance of the procedures, benefits, and probable adverse events associated with the protocol. Study subjects provided written informed consent, including permission to use the collected data for research purposes only.

### Subjects

**FIGURE 1:**
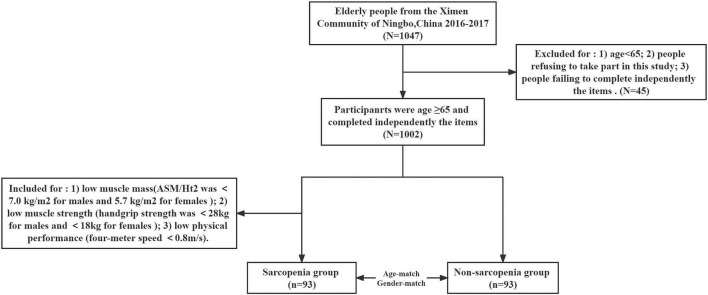
Flow diagram of this study.

Participants in the present study were chosen from the Ximen Community of Ningbo, China. There were 1,047 elderly people aged 65 years or more who were involved in our examination and completed a comprehensive geriatric assessment from November 2016 to March 2017. Inclusion criteria were the following: people aged 65 years old or more who were eligible to participate and people who can independently finish a comprehensive geriatric assessment, including tests of walking speed, grip strength, and muscle mass. Exclusion criteria were the following: people refusing to take part in this study; people failing to complete the items that they were required to be inspected independently; people who were aged < 65 years old. Based on the above criteria, participants with the record of hand strength, 4-m speed, or body composition (*n* = 28), or measurement of baPWV (*n* = 13), or the filled questionnaire (*n* = 4) were excluded. Thus, a total of 1,002 participants were analyzed. A total of 93 subjects with sarcopenia were diagnosed as having low muscle mass (ASM/Ht2 was <7.0 kg/m^2^ for males and <5.7 kg/m^2^ for females), low muscle strength (handgrip strength was <28 kg for males and <18 kg for females), and/or low physical performance (4-m walking speed < 0.8 m/s) by the Asian Working Group for Sarcopenia (AWGS) criteria. Accordingly, 93 non-sarcopenic subjects were screened in a paired design with 93 sarcopenic subjects of the same gender and similar age. The participants were further divided into sarcopenia group (*n* = 93) and non-sarcopenia group (*n* = 93) (details in Figure 1).

### Questionnaire

Data regarding sociodemographic and behavioral characteristics was obtained in a similar manner to our previous studies *via* face-to-face interactions. Sociodemographic variables, including age, gender, marital status, educational level, and occupation were assessed. The assessed social history included smoking habits (never, former smoker, or current smoker) and drinking habits (never, former drinker, occasional drinker, or everyday drinker). Former smokers and current smokers were asked how many cigarettes they smoked per day and how long they had smoked. We next obtained and assessed physical activity levels. The physical activity level was assessed using the short form of the International Physical Activity Questionnaire (IPAQ). A history of physical illness was established using standardized criteria that combined information from a questionnaire regarding the history of a physical illness, a physician’s diagnosis, and the corresponding medication or current or past medical treatment.

### Assessment of sarcopenia

Sarcopenia was diagnosed as low muscle mass, low muscle strength, and/or low physical performance by the Asian Working Group for Sarcopenia (AWGS) criteria. For low muscle mass, ASM/Ht2 was <7.0 kg/m^2^ for males and 5.7 kg/m^2^ for females, respectively. For low muscle strength, male handgrip strength was <28 kg, and female handgrip strength was <18 kg, respectively. Physical performance could be low with a 4-m walking velocity of 0.8 m/s.

The direct segmental multifrequency bioelectrical impedance analysis was used to analyze body composition features. Appendicular skeletal muscle mass (ASM) was summed up as the total skeletal muscle in the arms and legs. The relative skeletal muscle mass index (ASM/Ht2) was defined as ASM divided by height squared in meters. Muscle strength was collected to the nearest 0.1 kg with an accurate handgrip dynamometer. 4-m walking speed was tested on a straight corridor with a 6-m mark on the ground.

### CVD risk factors assessment

Based on previous research, the following are the three major risk factors for CVD: diabetes is defined as the use of hypoglycemic agents, a self-reported history of diabetes, or a FPG ≥ 7.0 mmol/L and/or OGTT 2hPG ≥ 11.1 mmol/L and/or HbA1c ≥ 6.5%; hypertension is defined as resting SBP ≥ 140 mmHg and/or DBP ≥ 90 mmHg and/or the self-reported current treatment for hypertension with antihypertensive medication; dyslipidemia is defined as using lipid-lowering drugs or having one or more of the following: TG ≥ 1.7 mmol/L, TC ≥ 5.18 mmol/L, HDL-C < 1.04 mmol/L, and LDL-C ≥ 3.37 mmol/L.

### Plasma sampling and RNA isolation

Fasting blood was collected in silicone-coated serum tubes with increased silica act clot activator within 1 h of collection in order to obtain plasma. Plasma and erythrocytes were separated by centrifuging blood samples at 845 g for 15 min at 4°C. Plasma was collected in aliquots into RNase/DNase-free tubes and stored at −80°C for further analysis. Total RNA was isolated from the plasma with a mirVana PARIS isolation kit (Ambion, Austin, TX, USA) according to the manufacturer’s instructions. To avoid discrepancies in results, all samples were extracted and analyzed in a single batch to minimize repeated freeze–thaw cycles of plasma samples. Briefly, total RNA was extracted from 400 μl of plasma. After adding the same volume of denaturing solution, the plasma miRNA level was normalized by adding 50 pmol/L of Caenorhabditis elegans miR-39 (cel-miR-39) as the peak control. All the samples were eluted with 100 μl of RNAse-free water.

### Quantification of circulating miRNA levels

For quantitative miRNA analysis, Bulge-LoopTM miRNA qPCR Primer Sets (RiboBio, Guangdong, China) were used to detect selected miRNAs expressions by quantitative reverse transcription polymerase chain reactions (qRT-PCRs) with iTaqTM Universal SYBR Green Supermix (BIO-RAD, Guangdong, China) in the 7900HT Fast Real-Time PCR System as previously reported. All qRT-PCR reactions were performed in triplicate, and the signal was collected at the end of every cycle. As there is no consensus on endogenous stable miRNAs in the circulation to act as house-keepers, the expression level of miRNAs in serum was normalized using spike-in cel-miR-39, which lacks sequence homology to human miRNAs.

### Statistical analysis

All other continuous variables were presented as means ± SD; classification variables were reported as percent-ages. Differences in baseline characteristics according to sarcopenia status were analyzed using *t*-tests, Pearson’s chi-square test, and Kruskal–Wallis rank tests. The associations between sarcopenia and CVRF were determined by analyzing each individual CVRF component or the total number of CVRF components. The simple logistic regression analysis was used to examine the independent influence of CVD risk factors on sarcopenia; the odds ratio (OR) and 95% (Confidence Interval, CI) were computed. In addition, adjustments for potential confounders such as age, gender, and BMI (model 1), residence, marital status, educational level, smoking status, drinking status, and income (model 2), and stroke, peptic ulcer, and kidney disease (model 3) were performed using multiple logistic regression analyses (Wald backward stepwise method). Analyses were repeated to determine the effect of individual CVRF components on sarcopenia components. The analysis generated regression coefficients, confidence interval, and Nagelkerke R2. Besides, the area under the receiver operating characteristic (ROC) curve and the Hosmer–Lemeshow goodness of fit statistic were calculated to assess the performance and calibration of the model, respectively. Through the analysis of qRT-PCR data, the 2^–ΔΔCt^ method was used to calculate the relative expression level for each miRNA, and the data was expressed as mean ± SD. An appropriate *t*-test was performed on the unpaired samples to analyze the miRNA changes. The Pearson’s method was used to conduct the correlation analysis between changes in circulating miRNAs and diagnostic indexes of sarcopenia (ASM/height2, handgrip strength, and 4-m velocity) as appropriate for data distribution. All the statistical analyses were performed using SPSS version 19.0, and *P*-values less than 0.05 were considered statistically significant.

## Results

### Characteristics of the study population

The cohort used in this study is the same as previously reported (32). A total of 186 elderly patients with clinically diagnosed sarcopenia and non-sarcopenia were enrolled in the final analysis of the study. The clinical and anthropometric characteristics of subjects by their sarcopenia status were presented in Table 1. The mean age of the sarcopenia and non-sarcopenia were 76.19 (±0.58) and 76.15 (±0.58) years, respectively. The height, weight, BMI, waist circumference, body fat, body muscle mass, and TG were lower in subjects who had sarcopenia compared to those without sarcopenia (*P* < 0.05). There were no statistically significant differences between the overall sarcopenic and non-sarcopenic groups in terms of marital status, residence status, educational status, income level, smoking status, drinking status, TC, LDL-C, HDL-C, and incidence of osteoporosis. Additionally, participants with sarcopenia had a statistically significant lower ASM, ASM/Height2, handgrip strength, 4-m velocity, and knee extension compared with participants without sarcopenia (*P* < 0.05). Hypertension and diabetes were more prevalent in the sarcopenia participants relative to the non-sarcopenia participants (*P* < 0.05).

**TABLE 1:** Clinical and anthropometric characteristics of study subjects according to the presence of sarcopenia.

Clinical parameters	Non-sarcopenia (*n* = 93)	Sarcopenia (*n* = 93)	*P*-value
Age (years)	76.19 ± 0.58	76.15 ± 0.58	0.639
Gender (M/F) Male, *n* (%)	34/59	34/59	
Height, cm	159.73 ± 0.78	156.03 ± 0.74	< 0.05
Weight (kg)	64.72 ± 1.19	52.97 ± 0.80	< 0.05
BMI (kg/m^2^)	25.32 ± 0.41	21.75 ± 0.29	< 0.05
Waist circumference (cm)	88.48 ± 10.11	78.73 ± 9.75	< 0.05
Body fat (%)	33.55 ± 0.72	31.71 ± 0.75	< 0.05
Body muscle mass (%)	64.72 ± 1.19	52.97 ± 0.80	< 0.05
TC (mmol/L)	4.42 ± 1.01	4.70 ± 1.06	0.589
TG (mmol/L)	1.68 ± 0.90	1.44 ± 0.62	< 0.05
LDL-C (mmol/L)	2.84 ± 0.96	3.04 ± 0.95	0.681
HDL-C (mmol/L)	1.48 ± 0.36	1.69 ± 0.46	0.130
Marital status			0.054
Yes, *n* (%)	60 (65%)	71 (76%)	
Others, *n* (%)	33 (35%)	22 (24%)	
Living with partner			0.129
Yes, *n* (%)	65 (70%)	74 (80%)	
No, *n* (%)	28 (30%)	19 (20%)	
Educational years			0.759
0–6, *n* (%)	35 (38%)	41 (44%)	
7–9, *n* (%)	32 (34%)	26 (28%)	
10–12, *n* (%)	16 (17%)	15 (16%)	
13 or more, *n* (%)	10 (11%)	11 (12%)	
Household income, *n* (%)			0.191
Lowest < 1000	0	0	
Lower middle 1,000–3,000	12 (13%)	5 (5%)	
Upper middle 3,000–5,000	27 (29%)	27 (29%)	
Highest > 5,000	54 (58%)	61 (66%)	
Smoking, *n* (%)			00.619
Never	72 (77%)	74 (80%)	
Former	17 (18%)	13 (14%)	
Current	4 (4%)	6 (6%)	
Alcohol drinking			00.472
Never, *n* (%)	51 (55%)	59 (63%)	
Former, *n* (%)	9 (10%)	8 (9%)	
Current, *n* (%)	32 (34%)	25 (27%)	
**Anthropometric indexes**			
ASM (kg)	17.31 ± 0.39	13.84 ± 0.28	< 0.05
ASM/Height^2^ (kg/m^2^)	6.71 ± 0.10	5.63 ± 0.07	< 0.05
Handgrip strength (kg)	24.18 ± 0.94	17.33 ± 0.55	< 0.05
4-m velocity (m/s)	1.12 ± 0.02	1.00 ± 0.03	< 0.05
Knee extension (kg)	19.93 ± 0.81	14.76 ± 0.59	< 0.05
Stroke, *n* (%)	5 (5%)	11 (12%)	0.117
Kidney disease, *n* (%)	9 (10%)	7 (2%)	0.601
Osteoporosis, *n* (%)	9 (9.7%)	10 (10.8%)	0.809
Peptic ulcer, *n* (%)	5 (5.3%)	9 (9.7%)	0.266
**Cardiovascular risk factors (CVRF)**			
Hypertension, *n* (%)	40 (43%)	54 (58%)	0.040
Diabetes, *n* (%)	38 (41%)	53 (57%)	0.028
Dyslipidemia, *n* (%)	49 (53%)	51 (55%)	0.769

### The relationship between sarcopenia and CVRF

The associations between the sarcopenia and the different CVRF components are present in Table 2. In the adjusted Model 1, the hypertension (but not diabetes and dyslipidemia) was found to be significantly associated with sarcopenia (*P* < 0.05). The OR and 95% CI for hypertension, which was significantly associated with sarcopenia, was 0.397 (0.200–0.789). After further adjusting for pertinent variables in Models 2–3, the CVRF components, including hypertension and dyslipidemia, were found to be significantly associated with sarcopenia (*P* < 0.05). For CVRF components, individuals with both hypertension [0.406 (0.182–0.906)] and dyslipidemia [0.415 (0.186–0.922)] were significantly associated with sarcopenia (*P* < 0.05), but not diabetes in the adjusted Model 3.

**TABLE 2:** Multiple logistic regression analysis of presence and components of CVRF for elderly patients with sarcopenia.

Variables	Univariate			Model 1			Model 2			Model 3		
	OR	95%CI	*P*	OR	95%CI	*P*	OR	95%CI	*P*	OR	95%CI	*P*
Presence of CVRF	0.392	0.161, 0.954	0.039	3.209	1.180, 8.729	0.022	0.294	0.096, 0.899	0.032	0.295	0.096, 0.902	0.032
**Components of CVRF**												
Hypertension	0.545	0.305, 0.975	0.041	0.397	0.200, 0.789	0.008	0.412	0.189, 0.896	0.025	0.406	0.182, 0.906	0.028
Dyslipidemia	0.917	0.515, 1.632	0.769	0.624	0.316, 1.231	0.174	0.424	0.192, 0.933	0.033	0.415	0.186, 0.922	0.031
Diabetes	0.521	0.291, 0.934	0.028	0.517	0.266, 1.008	0.053	0.607	0.289, 1.276	0.188	1.632	0.774, 3.440	0.198

Model 1: Adjustment for age, gender, and BMI; Model 2: Model 1 + adjustment for residence, marital status, educational level, smoking status, drinking status, and income; Model 3: Model 2 + adjustment for stroke, peptic ulcer, and kidney disease.

As shown in Table 3, the results of the applications of the models indicate that the increasing number of CVRF constituents affected sarcopenia. As the number of CVRF constituents increased, the risk of sarcopenia increased linearly. As a result of further covariate adjustment in Model 3, the OR and 95% CI for sarcopenia individuals with 1, 2, and 3 features of CVRF were 2.55 (0.83–7.82), 4.21 (1.34–13.23), and 8.16 (2.07–32.23) (*P*-values for the trends <0.001), respectively. It was showed that he prevalence of sarcopenia increased steeply with numbers of CVRF. Lastly, all models were evaluated for performance and calibration. All models performed well in terms of performance and calibration (area under the ROC curves > 0.80; Hosmer–Lemeshow goodness of fit *P* < 0.05).

**TABLE 3:** Regression analysis of the number of CVRF for elderly patients with sarcopenia.

Number of CVRF	Univariate		Model 1		Model 2		Model 3	
	OR (95%CI)	*P*	OR (95%CI)	*P*	OR (95%CI)	*P*	OR (95%CI)	*P*
1	1.61 (0.63, 4.12)	0.32	1.98 (0.69, 5.679)	0.21	2.50 (0.83, 7.54)	0.11	2.55 (0.83, 7.82)	0.10
2	2.69 (1.04, 6.94)	0.04	3.80 (1.28, 11.29)	0.016	4.30 (1.39, 13.32)	0.011	4.21 (1.34, 13.23)	0.014
3	2.92 (1.01, 8.491)	0.04	5.86 (1.64, 20.92)	0.006	9.25 (2.42, 35.27)	0.001	8.16 (2.07, 32.23)	0.003
*P* for trend	<0.018		<0.002		<0.001		<0.001	

Model 1: Adjustment for age, gender, and BMI; Model 2: Model 1 + adjustment for residence, marital status, educational level, smoking status, drinking status, and income; Model 3: Model 2 + adjustment for stroke, peptic ulcer, and kidney disease.

### Decrease in CVRF-associated circulating miR-29b in response to sarcopenia in the elderly

**FIGURE 2:**
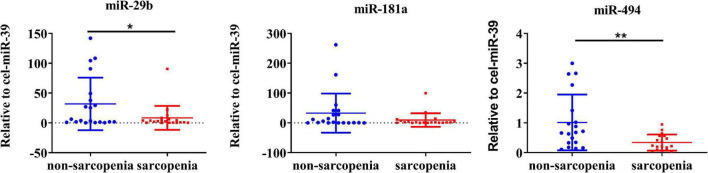
Changes in circulating microRNAs in response to sarcopenia in the elderly. The expression level of miRNAs was normalized using spike-in cel-miR-39; unpaired *T*-test was used for these data; *compared to non-sarcopenia of the elderly; *P < 0.05, **P < 0.01; *n* = 20.

**FIGURE 3:**
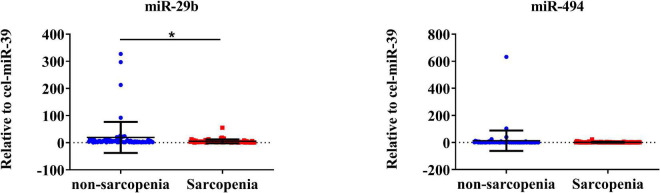
CVRF-associated circulating miR-29b decrease in response to sarcopenia in the elderly. The expression level of miRNAs was normalized using spike-in cel-miR-39; unpaired *T*-test was used for these data; *compared to non-sarcopenia of the elderly; *P < 0.05; *n* = 73.

The expression of CVRF-associated miRNAs [miR-29b (24, 25), miR-181a (26, 28), and miR-494 (29, 31)] were determined. As a result of a small-sample screening experiment, plasma miR-29b and miR-494 levels were significantly decreased in sarcopenia patients compared to those without sarcopenia. In contrast, miR-181a were not changed significantly (Figure 2). In order to verify that these miRNAs were differentially expressed, the experimental sample size was further extended through continuous analyses. It was also found that miR-29b levels decreased significantly in the sarcopenia group compared to the non-sarcopenia group. However, there were no significant differences in miR-494 levels between sarcopenic and non-sarcopenic groups (Figure 3).

### Correlation analysis between changes of miR-29b and ASM/height2, handgrip strength or 4-m velocity

**FIGURE 4:**
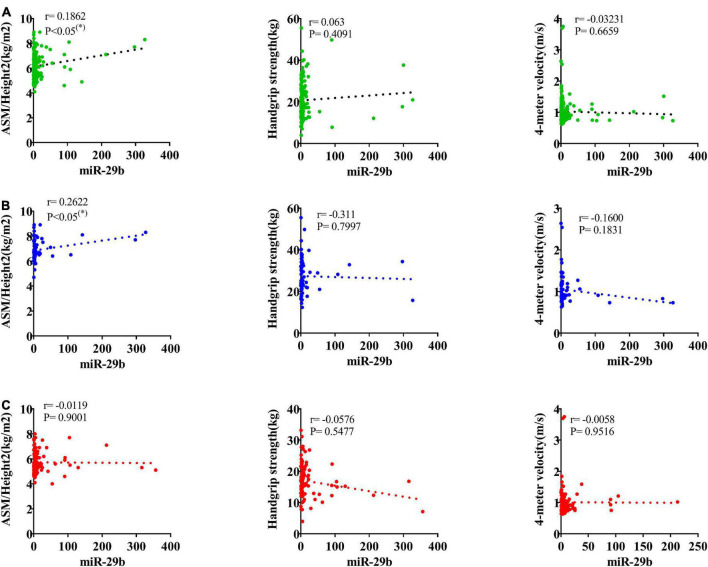
Correlation analysis between the changes of miR-29b and appendicular skeletal muscle mass (ASM)/Height^2^ (kg/m^2^), handgrip strength (kg), and 4-m velocity (m/s). The correlation between the changes of miR-29b and the diagnostic indicators of sarcopenia (ASM/Height2, handgrip strength, and 4-m velocity) in sarcopenic and non-sarcopenic subjects **(A)**. Pearson’s method was used for correlation analyses; *P < 0.05; *n* = 186. The correlation analysis of miR-29b with these indicators in male subjects **(B)**. Pearson’s method was used for correlation analyses; *P < 0.05; *n* = 67. The correlation analysis of miR-29b with these indicators in female subjects **(C)**. Pearson’s method was used for correlation analyses; *P < 0.05; *n* = 119.

**FIGURE 5:**
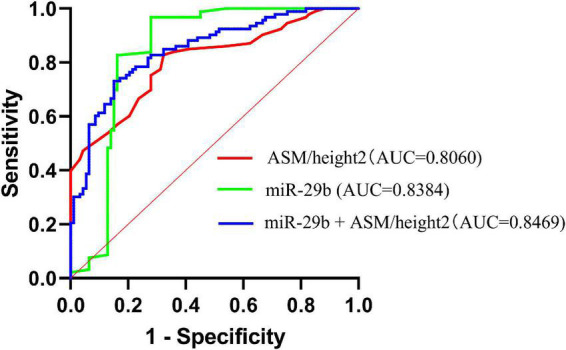
Receiver operating characteristic (ROC) curve was used to evaluate the diagnostic value of miR-29b, ASM/height2, and combine miR-29b with ASM/height2 for sarcopenia in the elderly; *n* = 186.

According to the AWGS definition, sarcopenia is characterized by low muscle mass, low muscle strength, as well as low physical strength. ASM/height2, handgrip strength, and 4-m velocity were used for the detection, and their levels were significantly decreased in elderly individuals with sarcopenia (Table 1). It was previously reported that miR-29b expression levels in plasma were significantly decreased in elderly patients with sarcopenia. Here, we investigated whether there was any correlation between expression level of miRNA-29b and the diagnostic indicators of sarcopenia (ASM/height2, handgrip strength, and 4-m speed) in sarcopenia group and non-sarcopenia group. There was a remarkable correlation between miR-29b and ASM/height2 (*r* = 0.1862, *P* < 0.05) (Figure 4A). In further analyses adjusted for gender, we also found that changes in the expression level of miR-29b were significantly associated with ASM/height2 in males, but not in females (*r* = 0.2622, *P* < 0.05) (Figures 4B, C). Finally, we have operated a ROC curve to evaluate the diagnostic value of miR-29b and ASM/height2 for sarcopenia in the elderly and found that miR-29b (ACU = 0.8384) was better than ASM/height2 (ACU = 0.8060) for the diagnosis of sarcopenia, and the combined test (ACU = 0.8469) was better than both alone for the diagnosis of sarcopenia (Figure 5).

## Discussion

This study was conducted to analyze the effects of varying CVRF component numbers on sarcopenia in China’s suburban-dwelling elderly population aged 65 and older, using the AWGS definition. We observed that sarcopenia prevalence correlated significantly with the number of CVRF components. As a result of the adjusted models, hypertension and dyslipidemia were significantly associated with an increased risk of sarcopenia. Then, we also found that plasma miR-29b levels were significantly downregulated in response to sarcopenia in elderly with CVRF. In particular, there was a remarkable correlation between miR-29b and ASM/height2.

Researchers are currently focusing on two aspects of sarcopenia and CVD. On the one hand, sarcopenia is directly associated with CVD, while on the other, sarcopenia is indirectly related to CVD by affecting CVRF. It has previously been suggested that the prevalence of sarcopenia is closely associated with increased risk factors and may itself be an individual risk factor for CVD (18, 33). In the cross-sectional study, we found that the prevalence of CVRF was significantly increased in the elderly Chinese populations with sarcopenia, including hyperlipidemia, dyslipidemia, and insulin resistance. Our results are largely consistent with these cross-sectional findings, although the criteria for diagnosing sarcopenia are significantly different. However, a prospective study conducted by the British Heart Research Center found that the risk of CVD in older men with sarcopenia was not significantly increased (34). In another study, it was found that sarcopenia was associated with lower risk factors of cardiovascular disease among obese, post-menopausal women (35). These contradictory results may be attributable to the various restrictions associated with the diagnosis of sarcopenia and CVRF, the disparities between study participants, and the varying ethnic characteristics. In fact, it was showed that the prevalence of sarcopenia varies from 0 to 48%, depending on the background, parameters, and cutoffs of the study population (2, 36–38). Moreover, there are significant regional differences in the prevalence of CVD, reflecting variances in CVRF between nations and regions. The prevalence of CVD is significantly reduced in developed countries such as France and Japan, which have the lowest age-standardized DALY rates for CVD. With a lower BMI, Asians have a higher risk of diabetes than Europeans, suggesting a difference between these two populations in terms of diabetes and its associated CVD risks (39–41). Thus, the potential mechanisms underlying the relationship between sarcopenia and CVRF are multifactorial, involving multiple pathophysiological changes in patients with sarcopenia, including muscle mitochondrial dysfunction, oxidative stress, hyperinflammatory states, microvascular endothelial dysfunction, and multiple metabolic disorders (insulin resistance), as well as similar lifestyle factors, such as poor nutrition and physical inactivity. In general, our findings provided the new evidence supporting the connection between sarcopenia and CVRF in elderly Chinese populations.

First, hypertension, one of the main chronic diseases of CVD, poses a serious threat to the health of humans (42). Obesity, overweight, smoking, and diabetes have been shown to be traditional independent risk factors for hypertension (19). However, 30% of hypertensive patients still lack these risk factors. To investigate whether sarcopenia is associated with hypertension, a cross-sectional study of the elderly in Korea found that the sarcopenia group had a higher prevalence of hypertension than the non-sarcopenia group (43). According to a Japanese study, women with sarcopenia are more likely to suffer from hypertension (44). Moreover, a study of more than 100 elderly Chinese people found that hypertension was closely related to the skeletal muscle mass of limbs in the elderly, and sarcopenia was a risk factor for hypertension in elderly men (45). In the present study, hypertension was associated with an elevated risk of sarcopenia, independently of age, gender, BMI, marital status, educational level, and smoking. The results of this study were consistent with those of previous studies. There are few studies on the relationship between sarcopenia and hypertension, and the underlying mechanisms between them are unclear. Sarcopenia was early thought to be associated with an increased incidence of hypertensive retinopathy and hypertensive kidney damage. It has recently been found that the development of hypertension is associated with a decrease in the number of capillaries surrounding muscle cells. It is speculated that blood pressure-induced changes in the capillary network of muscle tissue are a risk factor for sarcopenia in elderly patients.

Secondly, sarcopenia and dyslipidemia have been associated with only a few studies in Asia. In this study, although univariate analysis showed no correlation between sarcopenia and dyslipidemia, multivariate statistical analysis indicated that dyslipidemia was the second-most significant feature that was associated with sarcopenia after adjusting for confounding factors such as age, gender, BMI, marital status, educational level, and smoking, which is consistent with previous findings. According to a study from South Korea, the elderly with sarcopenia were at higher risk for dyslipidemia (46). In addition, dyslipidemia was also associated with sarcopenia in a 4-year longitudinal study of 538 elderly Japanese (47). The mechanism of dyslipidemia and its association with an accelerated reduction of muscle mass and strength remains unclear.

Finally, our study showed that there was no correlation between diabetes and sarcopenia. However, Srikanthan et al. reported that sarcopenia was associated with an increased risk of diabetes, and skeletal muscle is involved in insulin-mediated glucose absorption and is the main target of insulin action (48). Leenders et al. also found that both appendicular skeletal mass and leg extension strength were lower in individuals with type 2 diabetes mellitus compared with normoglycemic controls (49). The mechanism of diabetes and its association with sarcopenia is complex and remains unclear. It is likely that these contradictory results are due to variable susceptibilities within the studied population. There is a great variation in the study populations of these studies, resulting in a wide variation in sarcopenia rates. Besides that, it is also possible that this confounding arises from different proportions of patients receiving treatment.

As markers of cellular biological processes, changes in circulating miRNA levels can provide information about cells. In this study, selected miRNA-29b, miRNA-181a, and miR-484 are involved in diabetes and hypertension and/or clinical relevance, as they are closely related to CVD. Indeed, our study showed that plasma miR-29b levels were significantly downregulated in response to sarcopenia in elderly with CVRF. In this study, our results were similar to those found in previous studies. It was previously reported that exercise attenuates Ang II-induced muscle atrophy by suppression of miR-29b (50, 51). In addition, they demonstrated a novel engineered extracellular vesicle delivery system for miR-29b editing, which could be potentially used for muscle atrophy therapy (52, 53). Accordingly, miR-29b may be critical in the development of sarcopenia, though further research is necessary to determine exactly how it works. As previously reported in studies, sarcopenia is closely associated with CVRF, including diabetes, hypertension, and dyslipidemia. Our study is the first to show that the miRNA miR-29b, which is associated with CVRF, is decreased in sarcopenia response in the elderly, while miRNA-181a and miRNA-494 identified in this study were not changed, suggesting that sarcopenia and CVRF may be regulated by these miRNAs. However, there is few research to explore the common biomarker leading to sarcopenia and CVRF in the elderly. Thus, future studies are still needed to investigate the functions of miRNAs in patients with sarcopenia and CVRF.

According to AWGS criteria definition, sarcopenia is characterized by low muscle mass, low muscle strength, and/or low physical performance. ASM/height2, handgrip strength, and 4-m velocity are the current diagnostic criteria for sarcopenia (5). In the present study, we found that miR-29b expression levels were significantly correlated with ASM/height2. According to the majority of studies, the incidence of sarcopenia differs between older men and women (36). Accordingly, we further adjusted for gender and found that the expression level of miR-29b was significantly positively correlated with ASM/height 2 in males, but not in females. Mechanically, the decline in sex hormone levels may explain the difference in correlation of changes in miRNA levels with diagnostic indicators of sarcopenia between the sexes in our study.

There are still some limitations in our research. First, the study is a single-center clinical trial with a small sample size, and the results may need to be confirmed in a multicenter study with a larger sample size and area of coverage. The results would be more reliable if more patients of different races were included. Second, only three targeted miRNAs that were associated with CVRF in the literature were examined due to cost; thus, there may be other unstudied miRNAs that may be better indicators of CVD and sarcopenia status. Moreover, even though the study participants were relatively healthy, some of them may have certain diseases. The expression levels of miRNAs may be altered by disease conditions, such as degenerative diseases, malignancies, and autoimmune diseases.

In conclusion, we found that the prevalence of sarcopenia was significantly proportional to the number of CVRF components. In particular, hypertension and dyslipidemia were significantly associated with a higher risk of sarcopenia in the adjusted models. Therefore, the prevention and treatment of CVRF may be useful in preventing and delaying the onset of sarcopenia. A further prospective study is needed to establish the causality of CVRF components and sarcopenia. Moreover, our study has been proven that c-miRNAs may be considered as possible biomarkers for sarcopenia as a new diagnostic tool to monitor response to treatment. There is also a pressing need for further research on sarcopenia and CVRF to understand their relationship and mechanism. These can provide more evidence to develop potential interventions to improve clinical outcomes.

## Data availability statement

The raw data supporting the conclusions of this article will be made available by the authors, without undue reservation.

## Ethics statement

The studies involving human participants were reviewed and approved by the Ethics Committee of Ningbo No. 2 Hospital. The patients/participants provided their written informed consent to participate in this study. Written informed consent was obtained from the individual(s) for the publication of any potentially identifiable images or data included in this article.

## Author contributions

HY conceived and designed this study and revised the manuscript. NH and YLZ performed the experiments and collected and analyzed the data. NH, BF, ZZ, and YZ contributed the reagents, materials, and analysis tools that used in this study. NH wrote the manuscript. All authors contributed to the article and approved the submitted version.
